# Overexpression of PRDX2 in Adipose-Derived Mesenchymal Stem Cells Enhances the Therapeutic Effect in a Neurogenic Erectile Dysfunction Rat Model by Inhibiting Ferroptosis

**DOI:** 10.1155/2023/4952857

**Published:** 2023-02-08

**Authors:** Peng Chen, Zehong Chen, Jiancheng Zhai, Wende Yang, Hongbo Wei

**Affiliations:** Department of Gastrointestinal Surgery, The Third Affiliated Hospital of Sun Yat-sen University, 510630 Guangzhou, China

## Abstract

Neurogenic erectile dysfunction (NED) is a common and serious complication after pelvic surgery. The clinical translation of adipose-derived mesenchymal stem cell (ADSC) therapies in NED remains a major challenge due to their low survival rate and limited therapeutic effect. Peroxiredoxin 2 (PRDX2) is a member of the peroxidase family that exerts its therapeutic effects by inhibiting oxidative stress (OS) and ferroptosis, and PRDX2 is expected to enhance the therapeutic effect of ADSCs in treating NED. The purpose of this study was to investigate whether PRDX2 could improve the survival of ADSCs and determine whether overexpression of PRDX2 in ADSCs (PRDX2-ADSCs) could enhance the therapeutic effect of NED. This study investigated the potential role of PRDX2-ADSCs through a NED model induced by bilateral cavernous nerve injury (BCNI) and three in vitro models established by H_2_O_2_-stimulated ADSCs, H_2_O_2_-stimulated corpus cavernosum smooth muscle cells (CCSMCs), and RSL3-stimulated CCSMCs. We found that PRDX2 could significantly improve the viability of ADSCs by suppressing apoptosis and OS in H_2_O_2_-stimulated ADSCs. We also found that BCNI triggered ferroptosis of the corpus cavernosum, which was manifested by increased reactive oxygen species (ROS), total iron content, and MDA as well as decreased SOD and GSH. Our results further demonstrated changes in the expression of key proteins (GPX4 and ACSL4) in the ferroptosis pathway, whereas PRDX2-ADSCs ameliorated BCNI-induced erectile dysfunction and ferroptosis of the corpus cavernosum in NED rats. Consistently, PRDX2-ADSCs attenuated OS in H_2_O_2_-stimulated CCSMCs and inhibited ferroptosis in RSL3-stimulated CCSMCs, as evidenced by the decrease in ROS, total iron content, and MDA and the increase in SOD and GSH together with changes in ferroptosis-related protein (GPX4 and ACSL4) expression. In conclusion, overexpression of PRDX2 in ADSCs enhanced the therapeutic effect in a rat model of neurogenic erectile dysfunction by inhibiting ferroptosis via regulation of the GPX4/ACSL4 axis.

## 1. Introduction

Neurogenic erectile dysfunction (NED) is a common and serious complication after pelvic surgery such as radical prostatectomy and total mesorectal excision [1, 2]. Although remarkable progress has been made in surgical techniques and pelvic anatomy, especially the technique of preserving the pelvic autonomic nerve, erectile dysfunction (ED) may still occur in nearly 12.5% of patients with rectal cancer after radical surgery [3]. Phosphodiesterase type 5 inhibitors (PDE5Is) are widely known as first-line treatment drugs for ED, but they have limited therapeutic effect on NED [4–6]. Therefore, a more effective treatment for NED is urgently needed.

Mesenchymal stem cell (MSC) therapy is a new and potential treatment for many diseases [7–9]. It can be obtained from cord blood, dental pulp, bone marrow, adipose tissue, and other sources. Adipose tissue is rich in content and easy to obtain through minimally invasive surgery, and adipose-derived mesenchymal stem cells (ADSCs) secrete growth factors and immune regulatory factors more actively, which is ideal for cell therapy [10, 11]. However, the local oxidative stress (OS) microenvironment of the corpus cavernosum caused by cavernous nerve injury (CNI) impairs the survival and therapeutic effect of transplanted ADSCs, thus limiting their clinical application [12, 13]. Therefore, there is an urgent need for an effective method to improve the survival and therapeutic efficacy of ADSCs in the OS microenvironment in the corpus cavernosum.

Peroxiredoxin 2 (PRDX2) is a member of the peroxidase family that exerts its therapeutic effects by inhibiting OS and ferroptosis, and PRDX2 is expected to enhance the therapeutic effect of ADSCs in treating NED [14, 15]. However, whether PRDX2 can improve the survival of ADSCs and whether overexpression of PRDX2 in ADSCs (PRDX2-ADSCs) can improve NED by inhibiting ferroptosis remain to be investigated.

Ferroptosis, first reported by Dixon et al. in 2012, is a nonapoptotic form of cell death that depends on iron overload and lipid peroxidation [16]. It is characterized by iron accumulation [17], excessive production of lipid peroxides [18], and characteristic morphological changes in mitochondria under transmission electron microscopy [19]. Abundant studies have shown that ferroptosis imbalance can lead to various diseases, including neurodegenerative diseases [20], ischemia-reperfusion injury [21], and the occurrence of many tumors, such as colorectal cancer [22] and melanoma [23]. However, it is not clear whether ferroptosis is involved in the progression of NED.

To date, no literature on the effects of PRDX2 on ADSCs or NED is available. Accordingly, we investigated whether overexpression of PRDX2 in ADSCs could enhance the therapeutic effect in an NED rat model and explored the underlying processes.

## 2. Materials and Methods

### 2.1. Cell Identification and Culture

ADSCs were extracted from epididymal tissue of 4-week-old Sprague-Dawley (SD) male rats [24]. The cells were cultured in complete medium consisting of low-glucose Dulbecco's modified Eagle's medium (DMEM) (Gibco, USA), 10% fetal bovine serum (FBS) (Gibco, USA), and 1% penicillin-streptomycin solution (containing 100 U/mL penicillin and 100 *μ*g/mL streptomycin) at 37°C with 5% CO_2_.Then, the ADSCs at passage 3 were collected and incubated with antibodies against CD11b, CD29, CD34, CD44, CD45, and CD90 (all from eBioscience, USA) and detected by flow cytometry (BD FACS Canto, USA) [25]. As a previous study mentioned [24], the success of osteogenic and adipogenic differentiation demonstrated the pluripotency of ADSCs (Cyagen, China). Alizarin red staining and oil red O staining were used to identify osteoblasts and adipocytes, respectively. The initially extracted ADSCs were cultured for 3-5 days and then passaged and named ADSCs at passage 1. ADSCs at passages 3-5 were used in our subsequent experiments. Then, ADSCs were cultured in serum-free low-glucose DMEM containing different concentrations of H_2_O_2_ from 0 to 500 *μ*M for 24 h to construct cell injury and OS models in vitro.

Corpus cavernosum smooth muscle cells (CCSMCs) were extracted using a standard protocol described in a previous study [26]. In brief, the penile tissues were cut into 1-2 mm^3^ pieces and placed in an orderly manner in a T25 flask containing high-glucose DMEM (Gibco, USA) complete medium supplemented with 10% FBS (Gibco, USA) and 1% penicillin-streptomycin solution (Gibco, USA) (containing 100 U/mL penicillin and 100 *μ*g/mL streptomycin). CCSMCs grew from penis fragments and were passaged. The cells were identified by using immunofluorescence of calponin 1 antibody (Santa Cruz Biotechnology, USA). CCSMCs were used in experiments at passages 3-5. To construct CCSMC injury and OS models in vitro, the cells were cultured in serum-free high-glucose DMEM containing different concentrations of H_2_O_2_ from 0 to 800 *μ*M for 4 h.

ADSCs and CCSMCs were cocultured by using a transwell system with a 0.4 *μ*m polycarbonate membrane (Costar, Corning, NY, USA) as previously described [27, 28]. First, CCSMCs were planted in the lower chamber and then ADSCs were planted in the upper chamber. In group 3 (vehicle), ADSCs transfected with empty vehicle (vehicle) were cocultured with CCSMCs. In group 4 (ADSC-P), PRDX2-ADSCs were cocultured with CCSMCs.

### 2.2. Lentivirus Construction and Transfection

Lentivirus expressing rat PRDX2 was purchased from Hanbio Biotechnology (Shanghai, China) by using HBLV-r-PRDX2-3xflag-ZsGreen-PURO. The PRDX2 lentivirus was transfected into passage 2 ADSCs at a multiplicity of infection (MOI) of 100. Puromycin (2 *μ*g/mL) was used to screen ADSCs transfected with PRDX2. After 3 days of screening, we observed green fluorescence by fluorescence microscopy, which indicated that PRDX2 was successfully transfected into ADSCs. In addition, western blotting was performed to detect the expression of PRDX2. After identification, the cells were passaged for subsequent experiments.

### 2.3. Cell Proliferation Assays

Cell viability was tested by CCK-8 assay (Beyotime Biotechnology, China) according to the manufacturer's protocol. ADSCs or CCSMCs were seeded into 96-well culture plates overnight, and 10% CCK-8 solution was added. After 2 h, a microplate reader (BioTek Eon, USA) was used to detect the absorbance at 450 nm.

### 2.4. Apoptosis Assays

ADSCs were collected as previously described. Cell apoptosis was assessed by the Annexin V-APC/7-AAD Apoptosis Kit (E-CK-A218, Elabscience, China) according to the manufacturer's instructions. Cell apoptosis was evaluated by flow cytometry (BD FACS Canto, USA).

### 2.5. Detection of Intracellular ROS, Superoxide Anion, and Mitochondrial Superoxide

According to the manufacturer's protocol, intracellular ROS, superoxide anion, and mitochondrial superoxide were determined with DCFH-DA (Solarbio, China), DHE (BestBio, China), and MitoSOX (Invitrogen, USA), respectively. Fluorescence intensity was observed by fluorescence microscopy (Nikon, Japan) or detected by flow cytometry (BD FACS Canto, USA).

### 2.6. Animals and Experimental Design

Four-week-old male SD rats and ten-week-old male SD rats were purchased from the Experimental Animal Center of South China Agricultural University and housed in the Animal Experimental Center of South China Agricultural University. Animal experiments were approved by the Ethics Committee of Experimental Animals of South China Agricultural University (2021d117).

To assess the therapeutic effect of PRDX2-ADSCs on NED in vivo, a bilateral cavernous nerve injury (BCNI) model of SD rats was established by crushing the bilateral cavernous nerve (CN) 2-5 mm from the main pelvic ganglion (MPG) for 2 min [29]. Twenty-four ten-week-old male SD rats were randomly divided into four groups. The rats in the sham operation group only underwent laparotomy. SD rats of the other three groups received BCNI and bilateral intracavernous injection of phosphate-buffered saline (PBS) (100 *μ*L; PBS group), vehicle-ADSCs (1 × 10^6^ vehicle-ADSCs in 100 *μ*L PBS; vehicle group) or PRDX2-ADSCs (1 × 10^6^ PRDX2-ADSCs in 100 *μ*L PBS; ADSC-P group). Meanwhile, to trace the survival of transplanted cells in vivo, six male SD rats were divided into two groups. The two groups received BCNI surgery and were injected with vehicle-ADSCs (1 × 10^6^ vehicle-ADSCs in 100 *μ*L PBS; vehicle group) or PRDX2-ADSCs (1 × 10^6^ PRDX2-ADSCs in 100 *μ*L PBS; ADSC-P group). One animal in each group was sacrificed on the 3rd, 7th, and 14th days after the BCNI procedure and cell treatment.

### 2.7. Erectile Function Assessment

Two weeks after surgery, intracavernous pressure (ICP) and mean arterial pressure (MAP) were recorded to evaluate the erectile function of the animals. After anesthesia, the right carotid artery was exposed through a median incision of the neck, and a heparinized 24-G cannula was inserted into the right carotid artery to record MAP. At the same time, a heparinized 25-G butterfly needle was inserted into the penis root to measure ICP. To induce erection, the CN was stimulated with electrical stimulation, and its parameters were 1.5 mA, 20 Hz, and 50 s duration. All data were recorded by a BL-420s biological function system (Chengdu Taimeng Technology Ltd., China). Subsequently, penis tissues were collected for further evaluation.

### 2.8. Fluorescence Staining and Masson's Trichrome Staining

According to the manufacturer's protocol, superoxide anion and mitochondrial superoxide in the corpus cavernosum were detected with dihydroethidium (Beyotime Biotechnology, China) and MitoSOX (Invitrogen, USA). DAPI was used to stain the cell nuclei.

For immunofluorescence staining, penile sections were incubated with primary antibodies against SMA (1 : 200, Affinity Biosciences, AF1032), GPX4 (1 : 200, ABclonal, A1933), and ACSL4 (1 : 300, Affinity Biosciences, DF12141). Secondary antibodies included DyLight 488- (1 : 300, Affinity Biosciences, S0008), 594- (1 : 300, Affinity Biosciences, S0006), and CY3- (1 : 300, Affinity Biosciences, S0011) conjugated antibodies. Nuclei were stained with DAPI. Images were acquired using a fluorescence microscope (Nikon, Japan) or a confocal laser scanning microscope (Zeiss LSM 880, Germany).

Masson's trichrome staining was performed according to the standard protocol [30]. The ratio between smooth muscle and collagen in the corpus cavernosum reflects fibrosis of the corpus cavernosum. ImageJ 1.46 (National Institutes of Health, USA) was used for quantitative analysis of the images.

### 2.9. Western Blotting

Proteins in penile tissues and cells were extracted by using a protein extraction reagent containing RIPA buffer (Beyotime Biotechnology, China) and protease inhibitors (Beyotime Biotechnology, China). Protein concentrations in tissue and cell lysates were detected using a BCA Kit (Beyotime Biotechnology, China), and electrophoresis was performed. After electrophoresis, proteins were transferred to polyvinylidene fluoride membranes and incubated overnight at 4°C with the following primary antibodies: PRDX2 (1 : 1000, Abcam, ab109367), SMA (1 : 500, Affinity Biosciences, AF1032), GPX4 (1 : 1000, ABclonal, A1933), ACSL4(1 : 1000, Affinity Biosciences, DF12141), GAPDH (1 : 5000, ABclonal, AC001), and *β*-actin (1 : 1000, Cell Signaling Technology, D6A8). After incubation with secondary antibodies, including goat anti-rabbit IgG (H+L) HRP (1 : 5000, Affinity Biosciences, S0001), and goat anti-mouse IgG (H + L) HRP (1 : 5000, Affinity Biosciences, S0002) at room temperature, images were acquired using a Tanon 5200 (Tanon Science & Technology Co, Ltd, China) and measured by ImageJ 1.46 software (National Institutes of Health, USA).

### 2.10. OS Level and Iron Content Detection

The levels of intracellular malondialdehyde (MDA) and superoxide dismutase (SOD) represented the oxidative and antioxidant abilities, respectively. MDA and SOD in CCSMCs and the corpus cavernosum were detected with an MDA assay kit (Beyotime Biotechnology, China) and SOD assay kit (Beyotime Biotechnology, China) following the manufacturer's protocols. The content of iron in CCSMCs and the corpus cavernosum was detected by an iron assay kit (Nanjing Jiancheng Bioengineering Institute, A039-2-1, China) following the manufacturer's instructions. The absorbance was detected by a microplate reader (BioTek Eon, USA).

### 2.11. GSH Assay

Glutathione (GSH) was determined by assay kits following the manufacturer's protocols. The absorbance was detected by a microplate reader (BioTek Eon, USA).

### 2.12. Statistical Analysis

All data are expressed as the mean ± standard deviation, and GraphPad Prism V.9.0 (GraphPad Software, Inc., USA) was used to plot the graphs. Student's unpaired *t* test was used to compare the differences between the two groups. One-way analysis of variance (ANOVA) followed by Tukey's post hoc test was used to identify significant differences among groups. All data were repeated at least three times. *P* < 0.05 was considered statistically significant.

## 3. Results

### 3.1. Characterization of ADSCs and PRDX2-ADSCs

**Figure 1:**
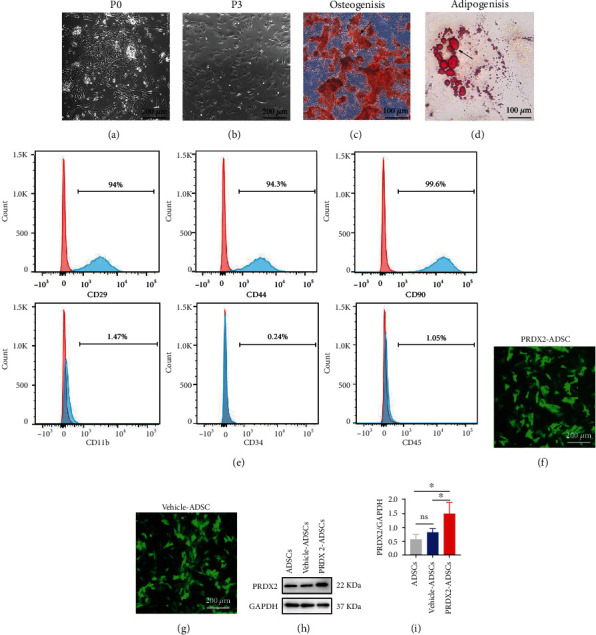
Characterization of ADSCs and PRDX2-ADSCs. (a, b) Morphology of ADSCs in passage 0 and passage 3. (c, d) Osteogenic and adipogenic differentiation assays of ADSCs. Representative images of ADSCs induced to differentiate into osteocytes (stained with alizarin red S) and adipocytes (stained with oil red O). (e) Flow cytometric analysis of surface markers on ADSCs. (f, g) Immunofluorescence analysis of the GFP expression in PRDX2-ADSCs and vehicle-ADSCs. (h, i) Western blot analysis showed that the level of PRDX2 markedly increased in transfected ADSCs (*n* = 3). ^∗^*P* < 0.05.

Primary ADSCs had a typical whirlpool and fibroblast-like morphology, and ADSCs at passage 3 had the same characteristics (Figures 1(a) and 1(b)). To evaluate the pluripotency of ADSCs, we performed osteogenic and adipogenic differentiation. The results showed that ADSCs could differentiate into osteoblasts and adipocytes (Figures 1(c) and 1(d)). Meanwhile, the surface markers of ADSCs were evaluated by flow cytometry. The results showed that ADSCs expressed the mesenchymal markers CD29 (94%), CD44 (94.3%), and CD90 (99.6%) but not endothelial or hematopoietic markers (CD11b (1.47%), CD34 (0.24%), and CD45 (1.05%)) (Figure 1(e)).

After transfection, fluorescence microscopy showed that most cells expressed green fluorescent protein, which means that ADSCs transfected with empty vector (vehicle-ADSCs) and ADSCs transfected with the PRDX2 gene (PRDX2-ADSCs) were successfully constructed (Figures 1(f) and 1(g)). Consistent with the fluorescence microscopy results, western blot results showed that the expression of PRDX2 in the PRDX2-ADSC group was significantly higher than that in the vehicle-ADSC and ADSC groups (Figures 1(h) and 1(i)).

### 3.2. PRDX2 Exerts Antiapoptotic and the Antioxidant Stress and Promotes Proliferation in ADSCs Exposed to H_2_O_2_ In Vitro

**Figure 2:**
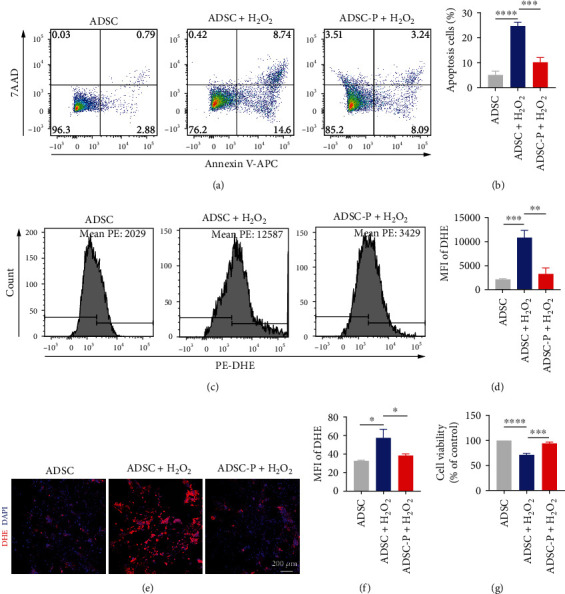
PRDX2 exerts antiapoptotic and the antioxidant stress and promotes proliferation in ADSCs exposed to H_2_O_2_ in vitro. (a, b) Apoptosis in ADSCs and PRDX2-overexpressing ADSCs cultured under H_2_O_2_ stimulation was analyzed using Annexin V-APC/7-AAD staining and flow cytometry (*n* = 3). (c, d) Intracellular ROS levels in ADSCs and PRDX2-overexpressing ADSCs cultured under H_2_O_2_ stimulation were evaluated using DHE-ROS and flow cytometry (*n* = 3). (e, f) Intracellular ROS levels in ADSCs and PRDX2-overexpressing ADSCs cultured under H_2_O_2_ stimulation were determined using DHE-ROS and fluorescence microscope (*n* = 3). (g) Effect of PRDX2 overexpression on ADSC viability by CCK-8 assay (*n* = 3). ADSC-P, ADSCs transfected with PRDX2. ^∗^*P* < 0.05, ^∗∗^*P* < 0.01, ^∗∗∗^*P* < 0.001, and ^∗∗∗∗^*P* < 0.0001. MFI: mean fluorescence intensity.

As shown in Figure S1a, compared with the control group (0 *μ*M H_2_O_2_), after treatment with 100, 200, 300, 400, and 500 *μ*M H_2_O_2_ for 24 hours, the survival rate of ADSCs decreased significantly when exposed to 400 *μ*M H_2_O_2_ (*P* < 0.05). Then, the concentration of 400 *μ*M H_2_O_2_ was determined to be the appropriate dose for further study (Figure S1a). To explore the cytoprotective effect of PRDX2 transfection on ADSCs, the cells were treated with H_2_O_2_ (400 *μ*M) for 24 h to construct an in vitro cell injury model of ADSCs. The antiapoptotic effect of PRDX2 transfection was evaluated by flow cytometry. The ADSC-P group inhibited ADSC apoptosis when treated with H_2_O_2_ (Figures 2(a) and 2(b)).

Next, the antioxidant stress effect of PRDX2 transfection was evaluated by flow cytometry and fluorescence microscopy. The flow cytometry results showed that the production of superoxide anions in the ADSC-P group was significantly reduced compared with that in the ADSC group when exposed to H_2_O_2_ (Figures 2(c) and 2(d), *P* < 0.05). Meanwhile, fluorescence microscopy showed that when exposed to H_2_O_2_, the production of superoxide anions in the ADSC-P group was significantly reduced compared with that in the ADSC group when exposed to H_2_O_2_ (Figures 2(e) and 2(f), *P* < 0.05). The CCK-8 assay indicated that the ADSC-P group had significantly increased ADSC viability compared with the ADSC group when exposed to H_2_O_2_ (Figure 2(g), *P* < 0.05). Together, these results suggested that overexpression of PRDX2 in ADSCs could reduce cell apoptosis and inhibit the production of superoxide anions to prevent cell damage, thus promoting the proliferation and survival of ADSCs.

### 3.3. PRDX2-ADSCs Alleviate H_2_O_2_-Induced Oxidative Stress in CCSMCs

**Figure 3:**
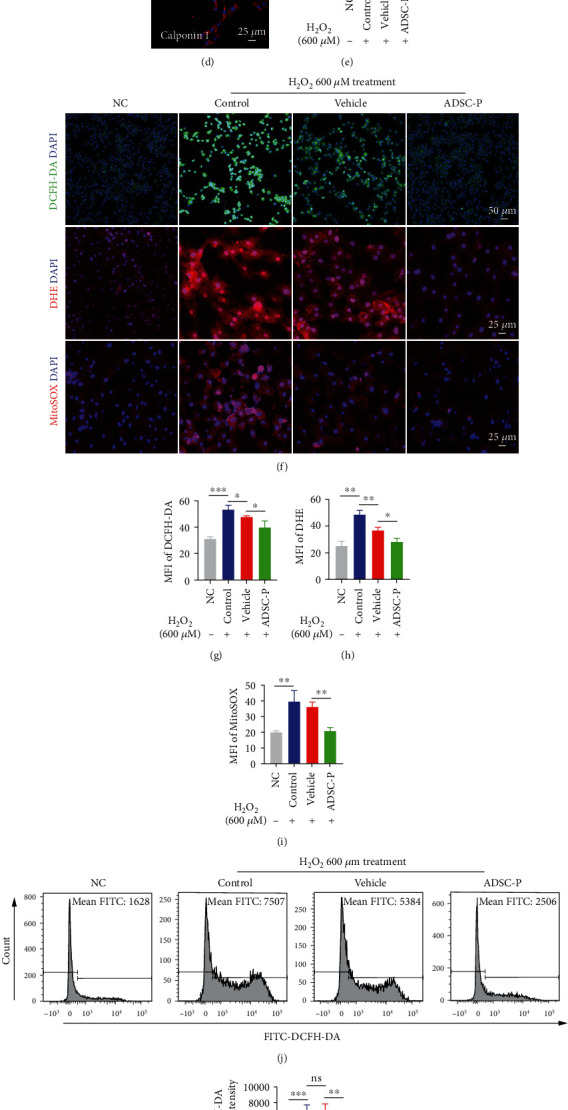
PRDX2 overexpression in ADSCs alleviates H_2_O_2_-induced CCSMC apoptosis and oxidative stress. (a) The diagram of the coculture system of ADSCs and CCSMCs. (b, c) Morphology of CCSMCs in passage 0 and passage 3. (d) Immunofluorescence of calponin 1 to identify CCSMCs. (e) The proliferation of CCSMCs cocultured with vehicle-ADSCs or PRDX2-ADSCs under H_2_O_2_ stimulation was analyzed by CCK-8 assay (*n* = 3). (f–i) Intracellular ROS, superoxide anion, and mitochondrial superoxide levels of CCSMCs after coculture as in (e) were determined using DCFH-DA, DHE, MitoSOX, and fluorescence microscope (*n* = 3). (j, k) Intracellular ROS levels of CCSMCs after coculture as in D using DCFH-DA and flow cytometry (*n* = 3). NC, CCSMCs without any treatment. Control, CCSMCs treated with H_2_O_2_ treatment. Vehicle, CCSMCs treated with H_2_O_2_ treatment and cocultured with ADSCs transfected with empty vector. ADSC-P, CCSMCs treated with H_2_O_2_ treatment and cocultured with ADSCs transfected with PRDX2. ^∗^*P* < 0.05, ^∗∗^*P* < 0.01, ^∗∗∗^*P* < 0.001, and ^∗∗∗∗^*P* < 0.0001. MFI: mean fluorescence intensity.

To evaluate the inhibitory effect of PRDX2-ADSCs on OS in vitro, CCSMCs were cocultured with ADSCs (Figure 3(a)). Primary CCSMCs grew from the penile pieces after 3 days and had a whirlpool-like and spindle morphology (Figure 3(b)). CCSMCs at passage 3 showed a shuttle pattern (Figure 3(c)) and were identified by immunofluorescence of calponin 1 (Figure 3(d)). As shown in Figure S1(b), compared with the control group (0 *μ*M H_2_O_2_), after treatment with 200, 400, 600, and 800 *μ*M H_2_O_2_ for 4 hours, the survival rate of CCSMCs decreased significantly when exposed to 600 *μ*M H_2_O_2_ (*P* < 0.05). Then, the concentration of 600 *μ*M H_2_O_2_ was determined as the appropriate dose for further study (Figure S1(b)).

CCSMC dysfunction under OS has been shown to impair penis erection function [31, 32]. Therefore, we established a coculture model to investigate the effect of PRDX2-ADSCs on the oxidative stress of CCSMCs under H_2_O_2_ exposure. As shown in Figure 3, H_2_O_2_ decreased proliferation (Figure 3(e)) and increased intracellular ROS generation (Figures 3(f) and 3(g)), superoxide anion (Figures 3(f) and 3(h)), and mitochondria superoxide (Figures 3(f) and 3(i)), while the ADSC-P group significantly increased proliferation (Figure 3(e)) and decreased OS (Figures 3(f)–3(i), *P* < 0.05). Similar to the fluorescence results, flow cytometry showed that the ADSC-P group inhibited intracellular ROS production in CCSMCs (Figures 3(j) and 3(k)).

### 3.4. Tracing of Transplanted Cells In Vivo

Since vehicle-ADSCs and PRDX2-ADSCs carry the GFP gene, they can emit green light for tracing in a rat model. The vehicle-ADSCs and PRDX2-ADSCs could both be detected in the penis at 3 and 7 days after transplantation. However, at 14 days after transplantation, only PRDX2-ADSCs could be seen in the penis, and vehicle-ADSCs had vanished. These results indicated that the survival time of PRDX2-ADSCs was longer than that of ADSCs after transplantation (Figure S2).

### 3.5. PRDX2-ADSC Treatment Improved Erectile Function in NED Rats

**Figure 4:**
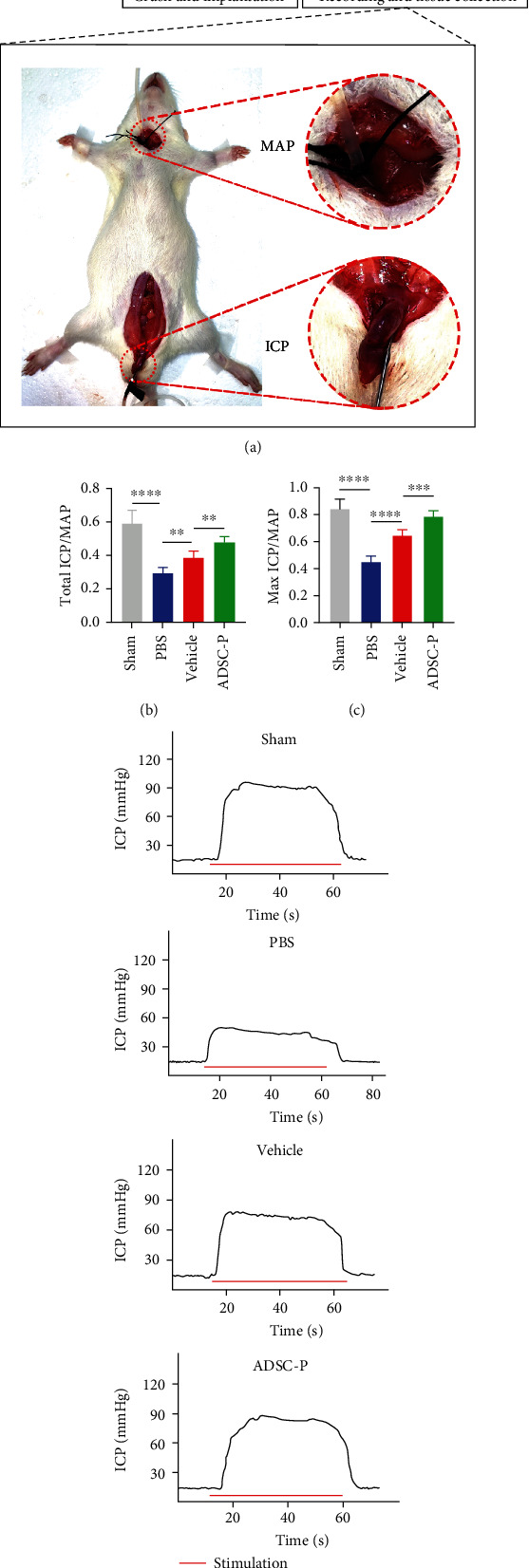
PRDX2-ADSC treatment improved erectile function in BCNI rats. (a) The schematic diagram of animal experiment. (b) ICP responses to electrostimulation in the sham, PBS, vehicle-ADSCs, and ADSC-P groups. (c, d) Quantitative analysis of total ICP/MAP and Max ICP/MAP. All values are depicted as mean ± SD from *n* = 6 animals per group. Vehicle, group injected with vehicle-ADSCs. ADSC-P, group injected with PRDX2-ADSCs. ^∗∗^*P* < 0.01, ^∗∗∗^*P* < 0.001, and ^∗∗∗∗^*P* < 0.0001.

The ratio of ICP to MAP induced by electrical stimulation of the CN in rats could represent the erectile function of rats (Figure 4(a)). Total ICP/MAP refers to the ratio of total ICP (area under the curve) to the mean MAP, while Max ICP/MAP refers to the ratio of maximum ICP to the mean MAP. The results showed that MAP did not differ significantly among the 4 groups. The results in the sham group showed that the ratios of total ICP (area under the curve) to MAP and maximum ICP to MAP were in the normal range. However, compared with the sham group, the PBS group showed significantly lower ratios of both ICP to MAP and maximum ICP to MAP (*P* < 0.05), indicating that the NED model was successfully constructed. Treatment in the vehicle and ADSC-P groups improved erectile function to varying degrees. Furthermore, compared with the vehicle group, the ADSC-P group was significantly better in terms of functional improvement, suggesting that the ADSC-P group could enhance the therapeutic effect of ADSCs in NED (Figures 4(b)–4(d)).

### 3.6. PRDX2-ADSC Treatment Prevented Fibrosis of the Corpus Cavernosum and Increased Cavernosal Smooth Muscle Content

**Figure 5:**
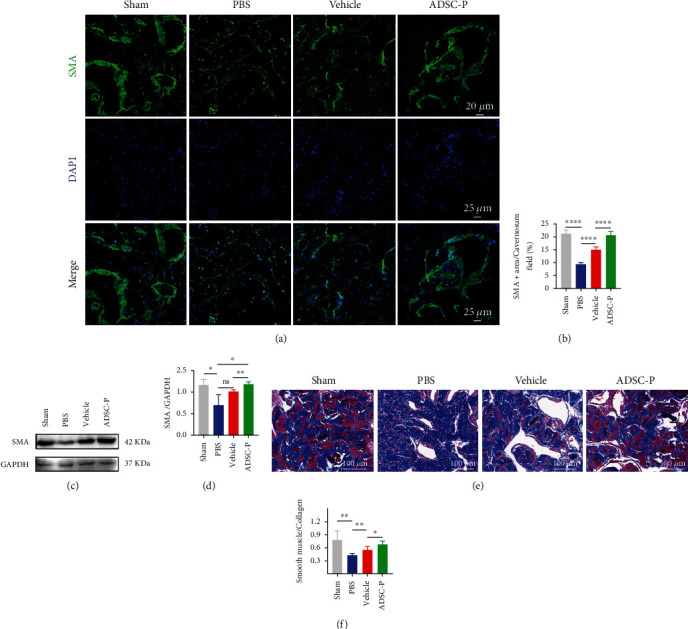
PRDX2-ADSC treatment prevented fibrosis of the corpus cavernosum and increased cavernosal smooth muscle content. (a, b) Immunofluorescent staining of SMA of corpus cavernosum in the sham, PBS, vehicle, and ADSC-P groups (*n* = 6 per group). (c, d) SMA protein expression of corpus cavernosum in the sham, PBS, vehicle, and ADSC-P groups determined by western blotting analysis (*n* = 3 per group). (e, f) Smooth muscle to collagen ratio in the corpus cavernosum assessed by Masson's trichrome staining in the sham, PBS, vehicle-ADSCs, and ADSC-P groups (*n* = 6 per group). Vehicle, group injected with vehicle-ADSCs. ADSC-P, group injected with PRDX2-ADSCs. ^∗^*P* < 0.05, ^∗∗^*P* < 0.01, ^∗∗∗^*P* < 0.001, and ^∗∗∗∗^*P* < 0.0001.

CNI-induced cavernous atrophy is one of the important mechanisms of NED. SMA immunofluorescence staining of penis reflected smooth muscle (SM) content, and the expression of SMA in the PBS group was significantly lower than that in the sham group (*P* < 0.05). However, the expression of SMA in the ADSC-P group and the vehicle group was significantly higher than that in the PBS group, and the ADSC-P group showed better improvement (Figures 5(a) and 5(b), *P* < 0.05). Western blotting results were generally consistent with SMA fluorescence, with one difference being that the SMA in the vehicle group was higher than that in the PBS group, but there was no statistically significant difference (Figures 5(c) and 5(d), *P* > 0.05). Masson's trichrome staining also showed that the ratio of SM to collagen in the PBS group was significantly lower than that in the sham group (Figures 5(e) and 5(f), *P* < 0.05). The ratio of SM to collagen in the ADSC-P group and the vehicle group was significantly increased, but the ADSC-P group showed further improvement (Figures 5(e) and 5(f), *P* < 0.05).

### 3.7. PRDX2-ADSC Therapy Prevents Oxidative Stress and Ferroptosis in the Corpus Cavernosum

**Figure 6:**
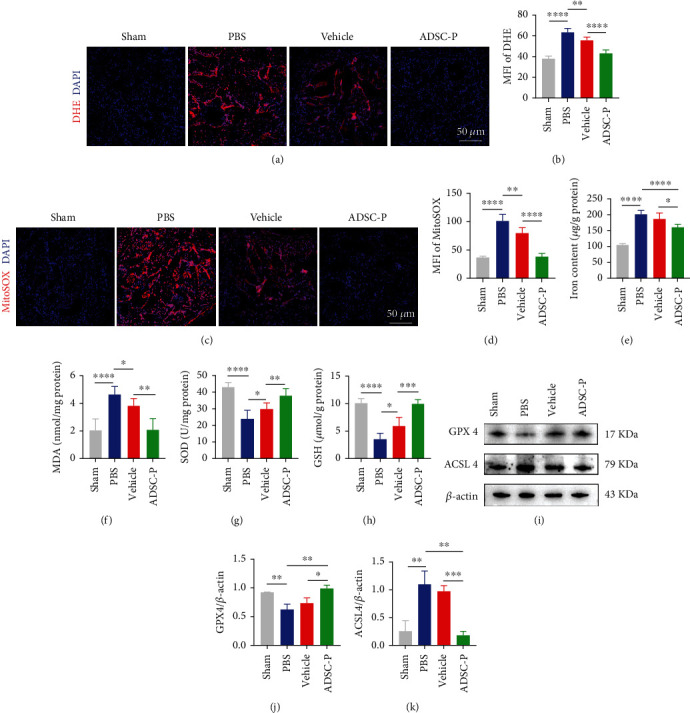
PRDX2-ADSC therapy prevented oxidative stress and ferroptosis injury in the corpus cavernosum. (a, b) Superoxide anion production was detected by fluorescence analysis (*n* = 6 per group). (c, d) Mitochondrial superoxide levels were detected by fluorescence analysis (*n* = 6 per group). (e-h) The levels of total iron content, MDA, SOD, and GSH in corpus cavernosum were measured (*n* = 6 per group). (i–k) Representative western blot results and quantification of GPX4 and ACSL4 in corpus cavernosum (*n* = 3 per group). Vehicle, group injected with vehicle-ADSCs. ADSC-P, group injected with PRDX2-ADSCs. ^∗^*P* < 0.05, ^∗∗^*P* < 0.01, and ^∗∗∗∗^*P* < 0.0001. MFI: mean fluorescence intensity.

CNI induced corpus cavernosum OS [33–35]; however, it decreased upon treatment with vehicle-ADSCs or PRDX2-ADSCs. The superoxide anion and mitochondrial superoxide levels in the corpus cavernosum were detected by DHE and MitoSOX staining. DHE and MitoSOX fluorescence showed that the production of ROS in the corpus cavernosum in the PBS group was significantly higher than that in the sham group but could be alleviated in the vehicle group and ADSC-P group (Figures 6(a)–6(d), *P* < 0.05). The ADSC-P group showed better improvement than the vehicle group (Figures 6(a)–6(d), *P* < 0.05). These results indicated that the ADSC-P group inhibited OS in the corpus cavernosum.

Recent studies have demonstrated that ferroptosis is related to the OS of CCSMCs in diabetes mellitus-induced ED and that human umbilical cord mesenchymal stem cells could treat diabetes mellitus-induced ED by inhibiting OS via attenuation of ferroptosis [36, 37]. Similarly, we were surprised to find that the total iron (Figure 6(e)) and MDA (Figure 6(f)) contents in the PBS group were significantly higher than those in the sham group (*P* < 0.05). In addition, the SOD (Figure 6(g)) and GSH (Figure 6(h)) contents in the PBS group were significantly lower than those in the sham group (*P* < 0.05). Treatment of the vehicle group reduced the iron contents (Figure 6(e), *P* > 0.05), and the ADSC-P group showed significant improvement compared with the vehicle group (Figure 6(e), *P* < 0.05). The vehicle group and ADSC-P group had significantly reduced MDA (Figure 6(f)) contents and increased SOD (Figure 6(g)) and GSH (Figure 6(h)) contents in the penis (*P* < 0.05). Meanwhile, the ADSC-P group showed further inhibition of OS than the vehicle group (Figures 6(f)–6(h), *P* < 0.05).

Then, we further explored the expression of key regulatory molecules in the ferroptosis regulatory pathway. The results of the PBS group showed that the expression of the positive regulator ACSL4 increased, while the expression of the negative regulator GPX4 decreased. Under the treatment of vehicle-ADSCs and PRDX2-ADSCs, the expression of GPX4 increased and the expression of ACSL4 decreased, but there was no significant difference in the vehicle group compared with the PBS group (Figures 6(i)–6(k)). Compared with the vehicle group, the ADSC-P group showed significant improvement (Figures 6(i)–6(k)). Based on the above results, we concluded that PRDX2-ADSC treatment can improve NED by inhibiting OS and ferroptosis.

### 3.8. PRDX2-ADSC Treatment Attenuated Ferroptosis in RSL3-Exposed CCSMCs by Regulating the GPX4/ACSL4 Axis

**Figure 7:**
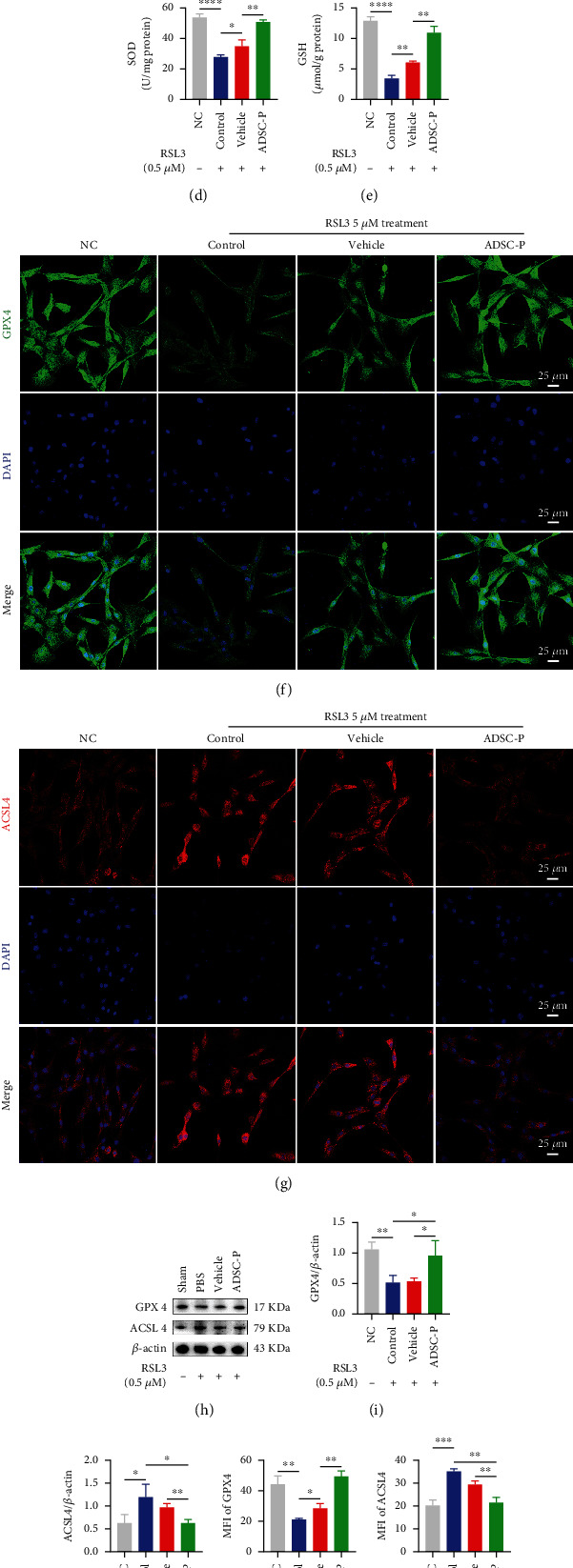
PRDX2-ADSC treatment attenuated ferroptosis in RSL3-exposed CCSMCs via GPX4/ACSL4 axis. (a) The proliferation of CCSMCs cocultured with vehicle-ADSCs or PRDX2-overexpressing ADSCs under RSL3 stimulation was analyzed by CCK-8 assay (*n* = 3). (b–e) The levels of total iron content, MDA, SOD, and GSH in CCSMCs of each group were measured by commercially assay kits (*n* = 3). (h–j) Representative western blotting results and quantification of GPX4 and ACSL4 in CCSMCs of each group (*n* = 3). (f, g, k, l) Representative fluorescence images and quantification of GPX4 and ACSL4 in CCSMCs of each group (*n* = 3). NC, CCSMCs without any treatment. Control, CCSMCs treated with RSL3 treatment. Vehicle, CCSMCs treated with RSL3 treatment and cocultured with ADSCs transfected with empty vector. ADSC-P, CCSMCs treated with RSL3 treatment and cocultured with ADSCs transfected with PRDX2. ^∗^*P* < 0.05, ^∗∗^*P* < 0.01, ^∗∗∗^*P* < 0.001, and ^∗∗∗∗^*P* < 0.0001. MFI: mean fluorescence intensity.

To further determine whether vehicle-ADSCs and PRDX2-ADSCs had specific inhibitory effects against ferroptosis, we studied the effects of vehicle-ADSCs and PRDX2-ADSCs on RSL3-induced ferroptosis in CCSMCs through a coculture system (Figure 3(a)). According to the previous literature and our own experimental results (Figure S1(c)), the concentration of 0.5 *μ*M RSL3 was determined to be the appropriate dose for further study [36]. RSL3, an inhibitor of GPX4, is widely considered an activator of ferroptosis. RSL3 treatment caused ferroptosis in CCSMCs and a decrease in cell viability (Figure 7(a)). However, after coculture with vehicle-ADSCs and PRDX2-ADSCs, the viability of CCSMCs increased, and the ADSC-P group improved significantly (Figure 7(a), *P* < 0.05). In RSL3-stimulated CCSMCs, the vehicle group and ADSC-P group inhibited RSL3-induced elevated total iron content, suppressed MDA levels, and increased SOD and GSH levels (Figures 7(b)–7(e)). Compared with the vehicle group, the ADSC-P group had a stronger inhibitory effect on ferroptosis (Figures 7(b)–7(e), *P* < 0.05).

**Figure 8:**
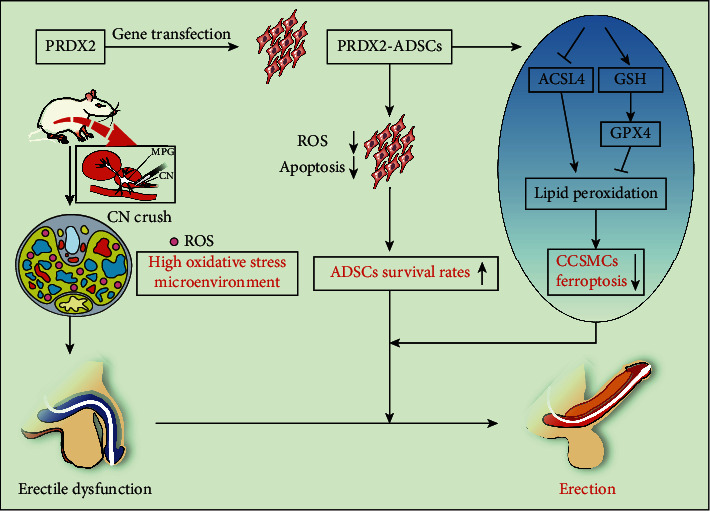
Schematic illustration of PRDX2-ADSCs for BCNI-ED treatment.

Then, we further explored the mechanism of PRDX2-ADSCs against ferroptosis in vitro. In RSL3-stimulated CCSMCs, the vehicle group and ADSC-P group exhibited increased expression of GPX4 and inhibited expression of ACSL4 (Figures 7(h)–7(j)). Compared with the vehicle group, the ADSC-P group showed stronger inhibition of the ferroptosis pathway (Figures 7(h)–7(j), *P* < 0.05). Similarly, the fluorescence of GPX4 and ACSL4 also showed that the vehicle group and ADSC-P group had an inhibitory effect on RSL3-induced ferroptosis of CCSMCs, but ADSC-P had a further inhibitory effect (Figures 7(f), 7(g), 7(k), and 7(l), *P* < 0.05). In conclusion, these results demonstrated for the first time that overexpression of PRDX2 in ADSCs improved their survival and inhibited the OS of CCSMCs through the attenuation of ferroptosis, thus enhancing therapeutic effects in NED (Figure 8).

## 4. Discussion

NED has a high incidence rate after pelvic radical surgery, which seriously affects the physical and mental health of patients [2, 38, 39]. It is a disease that is difficult to cure, and most traditional treatment methods show limited effectiveness [40, 41]. Stem cell therapy for NED is a promising therapeutic method [29, 42, 43]. However, it needs to be optimized prior to clinical application due to its low survival rate and limited therapeutic effect in the transplanted OS microenvironment. Therefore, the present study investigated whether the overexpression of PRDX2 in ADSCs could enhance the therapeutic effect of ADSCs on an NED rat model. We found that PRDX2-ADSC treatment enhanced the therapeutic effect in NED by increasing the survival of ADSCs and attenuating OS and ferroptosis in the corpus cavernosum.

The low survival rate and rapid apoptosis of MSCs after transplantation bring great challenges to their clinical translation [12, 13]. Therefore, the use of interventions to maximize the therapeutic potential of MSCs has aroused great interest. Cell transfection may be a powerful measure to tackle this challenge [28, 42]. Studies have reported that gene modification can increase the paracrine function of MSCs and their ability to resist harsh environments [28, 42]. Our results revealed that PRDX2 transfection could increase the proliferation of ADSCs and protect ADSCs from apoptosis and OS in vitro. A possible reason for this is that PRDX2 is an antioxidant enzyme that protects cells, including ADSCs by removing ROS [12, 13, 25, 44, 45].

Moreover, we observed that PRDX2-ADSCs could protect CCSMCs from OS in vitro. A possible reason for this is that PRDX2-ADSCs act through paracrine factors or exosomes. A previous study reported that the overexpression of HIF-1*α* in ADSCs can rescue endothelial cell dysfunction by enhancing the paracrine effect of ADSCs, thus treating diabetic foot [28]. In addition, ADSCs overexpressing Nrf2 enhanced the proliferation and angiogenesis of EPCs in a high-glucose environment by secreting Nrf2-rich exosomes, thus improving diabetic foot [46]. Consistent with previous studies, we also found that PRDX2-ADSCs may protect CCSMCs from OS through a paracrine mechanism.

SMA is a relatively abundant component in CCSMCs and is the structural basis of smooth muscle cell contraction and thus represents the smooth muscle content in the penis. Several studies have shown that CNI might lead to a decrease in CCSMCs [33, 35]. Consistent with previous studies, our NED model also showed that the SMA content in the PBS group was significantly lower than that in the sham group, and the SMA content was increased to different degrees after treatment with vehicle-ADSCs and PRDX2-ADSCs. Compared with that in the vehicle group, SMA expression in the ADSC-P group was higher. Meanwhile, the ratio of SM to collagen in the ADSC-P group was higher than that in the vehicle group. The reason for this may be that PRDX2-ADSCs inhibited OS in vivo.

In the current study, ADSCs could improve the fibrosis of CCSMC, similar to the findings of several studies [42, 43, 47]. BCNI can cause irreversible progression of penis tissue fibrosis through the deposition of extracellular matrix (ECM), which occurs in the early stage after injury [43]. Zhang et al. and Wu et al. reported that MSCs derived from human gingiva or ADSCs pretreated with lipopolysaccharide could inhibit the expression of ECM (collagen I/IV and fibronectin) proteins in the corpus cavernosum of BCNI rats, thereby improving penile fibrosis [43, 48]. In our study, Masson's trichrome staining showed that ADSCs and PRDX2-ADSCs have significant therapeutic effects on NED. In addition, PRDX2-ADSCs were more effective than ADSCs, which is reasonable. Some studies have shown that oxidative stress is closely related to penile tissue fibrosis and that inhibiting oxidative stress could improve penile tissue fibrosis, which is consistent with our findings [49–51].

Previous studies have reported that OS is the key pathophysiological mechanism of NED [52, 53]. Injury of the cavernous nerve can induce the production of OS in the corpus cavernosum, thereby significantly reducing the content of cavernous endothelial cells, smooth muscle cells, and cavernous nerves necessary for penis erection, thus leading to NED. Therefore, inhibition of ROS could significantly reduce cell death and improve NED. It has been reported that OS can induce various forms of cell death, such as apoptosis, pyroptosis, and ferroptosis. Some studies have reported that apoptosis of the corpus cavernosum in diabetic mice increases, but cell death can only be partially inhibited, and the improvement of ED is not obvious after the use of apoptosis inhibitors. These results suggested that apoptosis is not the only cause of cell death in the corpus cavernosum [52]. Several studies have reported that the injury of CCSMCs in erectile dysfunction caused by diabetes is related to ferroptosis, and human umbilical cord mesenchymal stem cells can improve erectile dysfunction in diabetic rats by reducing ferroptosis. However, the role of ferroptosis in NED remains unclear. In this study, we first found that PRDX2-ADSCs could reduce the total iron content and oxidative stress levels, including ROS, MDA, SOD, and GSH, both in vivo and in vitro. Then, we found that PRDX2-ADSCs could increase the expression of GPX4 and decrease the expression of ACSL4 both in vivo and in vitro. These results suggested that PRDX2 might enhance the therapeutic effect of ADSCs on NED by inhibiting the ferroptosis pathway of CCSMCs.

The current study has some limitations. First, experimental results obtained from rat models might not be fully representative of the therapeutic effect in patients. Second, this study only explored the therapeutic effect of PRDX2-ADSCs on NED rat model for 14 days at present; we will plan to extend different time points to observe the effect of cavernous nerve repair in future research.

## 5. Conclusions

In conclusion, our current investigation revealed that overexpression of PRDX2 in ADSCs enhanced the therapeutic effect on NED. Moreover, we demonstrated that overexpression of PRDX2 in ADSCs not only improved the survival of ADSCs by inhibiting apoptosis and OS but also inhibited the OS and ferroptosis of CCSMCs by regulating the GPX4/ACSL4 axis. These findings will provide potential therapeutic methods for NED and new insights for ADSCs in the mechanism of NED treatment.

## Data Availability

The data of the materials and methods and results to support the conclusions are included in this article. If any other data are needed, please contact the corresponding author.

## Supplementary Materials

Supplementary Materials Supplementary file 1. Supplementary Figure S1: exploration of optimum concentration of different activators. (a) Effects of various low doses of H_2_O_2_ on ADSC viability at 24 h (*n* = 6). (b) Effects of various low doses of H_2_O_2_ on CCSMCs viability at 4 h (*n* = 6). (c) Effects of various low doses of RSL3 on CCSMCs viability at 24 h (*n* = 6). Figure S2: tracing of transplanted cells in vivo. (a) Vehicle-ADSCs were detected in the penis on day 3 and day 7 after transplantation in rats with BCNI. (b) PRDX2-ADSCs were detected in the corpus cavernosum on day 3, day 7, and day 14 after transplantation in rats with BCNI.
