# Supplementary material for: Overexpression of PRDX2 in Adipose-Derived Mesenchymal Stem Cells Enhances the Therapeutic Effect in a Neurogenic Erectile Dysfunction Rat Model by Inhibiting Ferroptosis
Source: Oxid Med Cell Longev. 2023 Feb 8;2023:4952857. doi: 10.1155/2023/4952857 (PMC9931470; doi:10.1155/2023/4952857)
Supplement: Supplementary Materials — Supplementary file 1. Supplementary Figure S1: exploration of optimum concentration of different activators. (a) Effects of various low doses of H2O2 on ADSC viability at 24 h (n = 6). (b) Effects of various low doses of H2O2 on CCSMCs viability at 4 h (n = 6). (c) Effects of various low doses of RSL3 on CCSMCs viability at 24 h (n = 6). Figure S2: tracing of transplanted cells in vivo. (a) Vehicle-ADSCs were detected in the penis on day 3 and day 7 after transplantation in rats with BCNI. (b) PRDX2-ADSCs were detected in the corpus cavernosum on day 3, day 7, and day 14 after transplantation in rats with BCNI. [file 4952857.f1.docx]

Figure S1


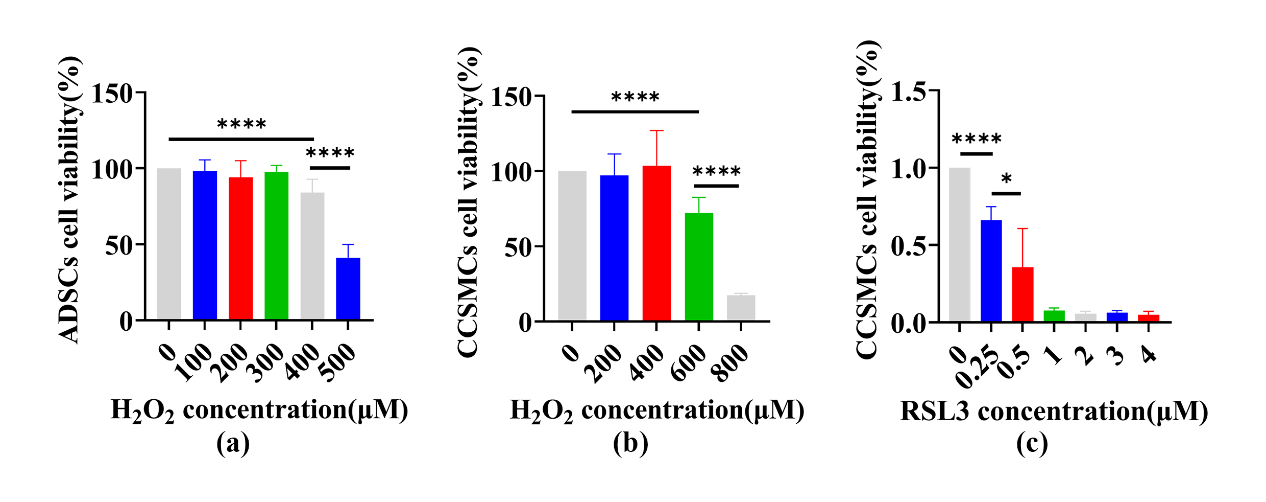


Figure S1: Exploration of optimum concentration of different activators. (a)Effects of various low doses of H_2_O_2_ on ADSC viability at 24h (n=6). (b) Effects of various low doses of H_2_O_2_ on CCSMCs viability at 4h (n=6). (c) Effects of various low doses of RSL3 on CCSMCs viability at 24h (n=6).

Figure S2


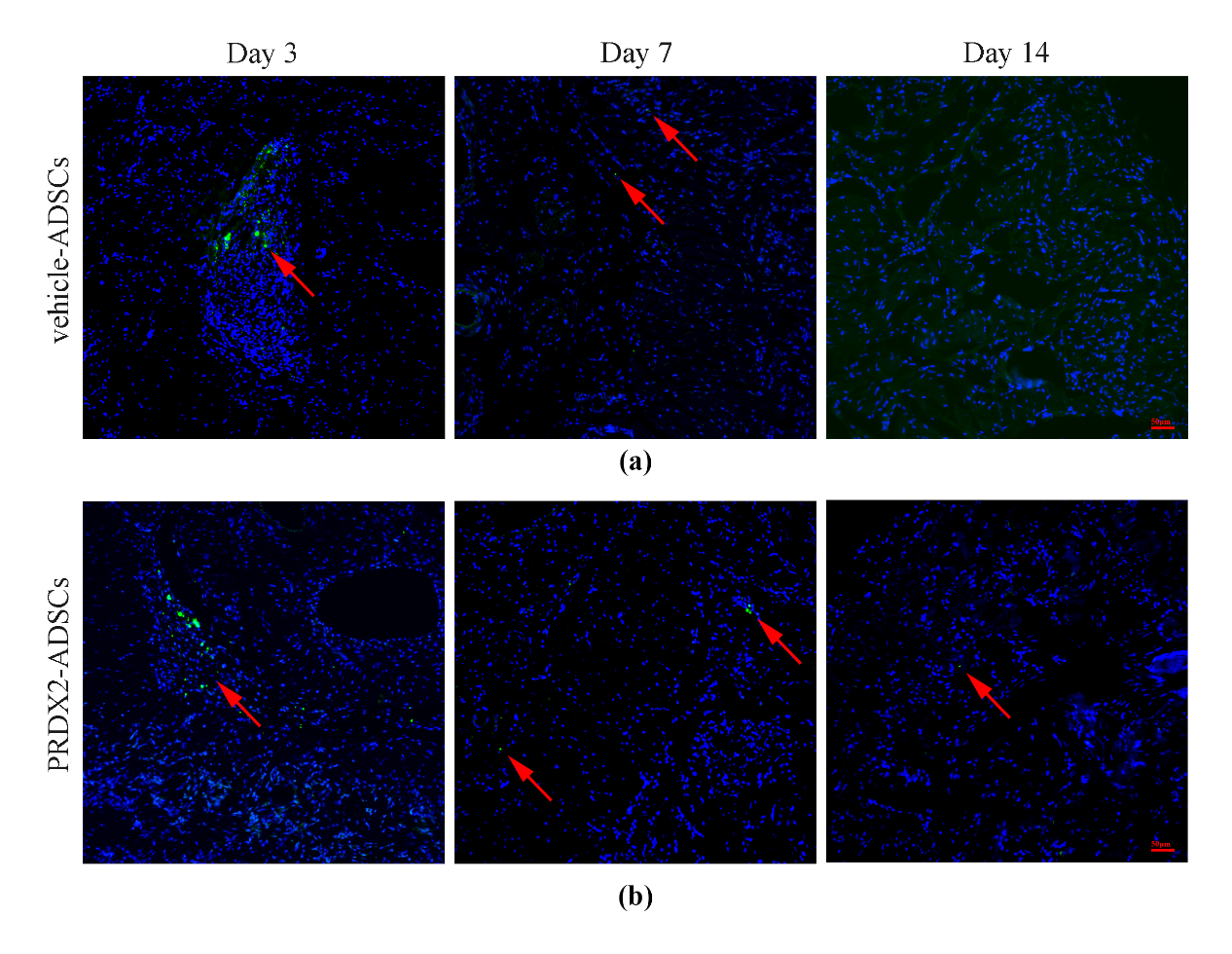


Figure S2: Tracing of transplanted cells in vivo. (a) Vehicle-ADSCs were detected in the penis on Day 3 and Day 7 after transplantation in rats with BCNI. (b) PRDX2-ADSCs were detected in the corpus cavernosum on Day 3, Day 7 and Day 14 after transplantation in rats with BCNI.
